# Brain Connectivity Changes During Bimanual and Rotated Motor Imagery

**DOI:** 10.1109/JTEHM.2022.3167552

**Published:** 2022-04-14

**Authors:** Jung-Tai King, Alka Rachel John, Yu-Kai Wang, Chun-Kai Shih, Dingguo Zhang, Kuan-Chih Huang, Chin-Teng Lin

**Affiliations:** Brain Research CenterNational Yang Ming Chiao Tung University Hsinchu 30010 Taiwan; CIBCI LaboratoryAustralian AI Institute, FEIT, University of Technology Sydney 1994 Ultimo NSW 2010 Australia; Department of Electronic and Electrical EngineeringUniversity of Bath 1555 Bath BA2 7AY U.K.; Department of Electrical and Computer EngineeringNational Yang Ming Chiao Tung University Hsinchu 30010 Taiwan

**Keywords:** Bimanual coordination, motor imagery, brain connectivity

## Abstract

Motor imagery-based brain-computer interface (MI-BCI) currently represents a new trend in rehabilitation. However, individual differences in the responsive frequency bands and a poor understanding of the communication between the ipsilesional motor areas and other regions limit the use of MI-BCI therapy. **Objective:** Bimanual training has recently attracted attention as it achieves better outcomes as compared to repetitive one-handed training. This study compared the effects of three MI tasks with different visual feedback. **Methods:** Fourteen healthy subjects performed single hand motor imagery tasks while watching single static hand (traditional MI), single hand with rotation movement (rmMI), and bimanual coordination with a hand pedal exerciser (bcMI). Functional connectivity is estimated by Transfer Entropy (TE) analysis for brain information flow. **Results:** Brain connectivity of conducting three MI tasks showed that the bcMI demonstrated increased communications from the parietal to the bilateral prefrontal areas and increased contralateral connections between motor-related zones and spatial processing regions. **Discussion/Conclusion:** The results revealed bimanual coordination operation events increased spatial information and motor planning under the motor imagery task. And the proposed bimanual coordination MI-BCI (bcMI-BCI) can also achieve the effect of traditional motor imagery tasks and promotes more effective connections with different brain regions to better integrate motor-cortex functions for aiding the development of more effective MI-BCI therapy. *Clinical and Translational Impact Statement* The proposed bcMI-BCI provides more effective connections with different brain areas and integrates motor-cortex functions to promote motor imagery rehabilitation for patients’ impairment.

## I. Introduction

Recent studies have reached a consensus on the feasibility of motor imagery-based brain-computer interface (MI-BCI) for stroke rehabilitation applications, especially in upper limb rehabilitation [1]–[3]. Motor imagery is the dynamic cognitive state achieved by rehearsing a motor action in the working memory without any overt motor output. In traditional MI-BCI therapy, patients perform one hand motor imagery tasks through electroencephalography signals (Mu and Beta sensorimotor rhythms, SMR) that communicate between the human brain and a computer to provide sensory feedback [1] or control the assistance device (e.g., exoskeleton) for muscle strength training [4], [5]. Patients exhibited significantly improved the upper limb function [6], motion-related neural activity in the ipsilesional site [7]–[9], and brain connectivity of the motor cortex [10] with MI-BCI therapy. Several researchers have also noted neuroplasticity changes and structural reorganization in the brain of stroke patients [11]–[13] after a period of training with MI-BCI. MI-BCI is known to repair and restore motor functions [6], [10], [11], [14], [15] by inducing changes in brain activity initiated by attempted movements of the affected hand, promoting motor recovery of remaining neurons and cortex reorganization in the ipsilesional motor areas. Nevertheless, some studies note that it can have a negative impact on motion recovery due to the plasticity of activated ipsilateral motion projections, competitive interactions, and compensatory movements after stroke [16]–[19]. As bilateral motion facilitates both spatial and time coordination [20], bilateral arm training (BAT) has been recently used to reconcile these problems. Bilateral arm training is known to enable (a) motor cortex disinhibition of the injured hemisphere, (b) enhanced paths from the contralesional region to recruitment, and (c) upregulation of descending motor neuron commands to propriospinal neurons [21], [22]. Although the effects of BAT are still debated [23], [24], few studies have studied the bimanual MI [25], [26] and its effect on the patients with movement deficits [27]–[29]. Bimanual movement was associated with power suppression in alpha, beta and gamma spectral bands. Even the activation pattern of bi-manual MI is difference for these patients, it still can be accurately classified and be used for restoration of motor function of highly disabled patients.

A major challenge that limits the use of MI-BCI therapy in stroke patients is the use of channel-based information to discriminate MI patterns. This is because the responsive frequency bands of MI-BCI are not consistent between subjects and within subjects [30], and main MI-BCI features and alpha (mu) rhythm event-related desynchronization (ERD)/synchronization (ERS) responses may be altered after stroke [31]. This instability is complex as the main ERD/ERS features for MI-BCI could occur in different experiment time intervals, on different frequency bands, and in different brain areas. Even though some machine learning approaches [32]–[36] can improve classification accuracy [37], [38], these characteristics make it difficult to extract EEG features for MI-BCI training [39], [40] and subsequent classification. A promising approach to handle this limitation is to consider the relationship between sources of brain signal using brain connectivity method that offers linchpin information about neural interactions related to MI and identifies reliable biomarkers in stroke rehabilitation to assess motor recovery [2], [41]–[43].

Although MI-BCI can improve the movement function of the ipsilesional motor cortex, brain connectivity analysis can provide more clear evidence to support the notion that performing the unilateral motor imagery tasks benefits the connections between the ipsilesional motor region and other brain areas.

In this study, we designed a bimanual coordination MI-BCI (bcMI-BCI) that incorporates the BAT concept to study brain connectivity when performing unilateral motor imagery tasks. In addition, we compare bcMI-BCI with another two conditions: traditional signal static hand and single hand with rotation MI-BCI for the differences in brain connectivity, analyzed by transfer entropy (TE) [44], which naturally merges directional and dynamic information.

This study will compare three different motor imagery tasks: (a) Traditional MI (tMI) (b) Rotation Movement MI (rmMI) and, (c) Bimanual Coordination MI (bcMI). For the results of this study, we would approve the effect of bcMI and rmMI by comparing with tMI using machine learning algorithms: common spatial pattern (CSP) for feature extraction and linear discriminant analysis (LDA) for MI classification. Furthermore, this study aims to explore brain connectivity while performing ipsilateral motor imagery tasks in tMI, rmMI, and bcMI. We hypothesized that brain connectivity analysis would reveal that the proposed novel rehabilitation approach of bimanual and rotated motor imagery effectively reorganizes neurons for motor function and reconnects other brain regions to achieve complete hand coordination function.

## II. Materials and Methods

### A. Participants

Fourteen healthy adults were recruited to participate the experiments (mean age 23.2 years, range 21–27 years). All subjects are right-handed with no mental or medical illnesses and no previous experience with MI-BCI. Before the experiment, they signed a consent form indicating that they have a clear understanding of the experimental protocol, which was approved by the Research Ethics Committee for Human Subject Protection, National Chiao Tung University.

### B. Experimental Design and Procedure

**FIGURE 1.**
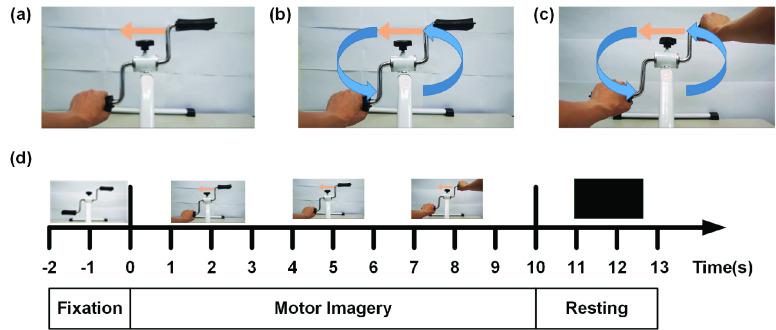
Three visual feedbacks for motor imagery tasks and the experimental paradigm. (a) Traditional MI task (tMI): assigned hand is static, (b) Rotation Movement MI task (rmMI): assigned hand performs the rotation movement in a forward fashion, (c) Bimanual Coordination MI task (bcMI): two hands perform the rotation movement in a forward fashion. The orange arrow points to the assigned hand for the motor imagery task. (d) The experimental procedure was divided into three stages, including the fixation stage (−2~0 s), motor imagery stage (0~10 s), and resting stage (10~13 s).

Three types of motor imagery tasks (as Fig. 1a-c) were designed to investigate differences in brain connectivity, including the (1) Traditional MI task (tMI), (2) Rotation Movement MI task (rmMI), and (3) Bimanual Coordination MI task (bcMI). As motor imagery and observation recruit the same brain regions, the subjects were asked to watch a video of the three conditions. In the tMI task, subjects performed motor imagery by imagining the rotation movement of the assigned hand while watching a video of the designated imagery hand statically holding one side of the hand pedal exerciser. The designated hand is indicated by an orange arrow in the middle of the exerciser. In the rmMI task, subjects imagined rotation movement of the assigned hand while watching a video of the designated imagery hand holding one pedal of the exerciser and performing rotation movements with it. In the bcMI task, subjects performed motor imagery by imagining the rotation movement of only the assigned hand while watching the video showing both hands holding each pedal of the exerciser to perform rotation movements. The assigned single hand was indicated by the yellow arrow in the video.

During the MI experiment, all participants sat in an electrically shielded cabin watching a 24” monitor from a 1-m distance. As shown in Fig. 1d, each trial started with the fixation stage (−2 to 0 sec) where the participants were instructed to look an image of a mini hand pedal exerciser in all three MI conditions. Then, a specific video of the MI tasks, played based on the MI condition of the trial, lasted for ten seconds and served as the motor imagery stage (0 to 10 s). During this motor imagery stage, subjects must imagine the rotation movement of single-hand assigned by the orange arrow in the top middle of the exerciser. Left and right arrows are randomly presented with the same probability. Then, a blank scenery screen is shown for three seconds as the resting stage (10 to 13s). The duration of each trial is 13 seconds (as Fig. 1d). For the three MI conditions, twenty trials are performed for each hand imagery and there were a total of 120 trials. The participants were instructed to remain still during the trial to reduce noise in the recorded EEG signal.

### C. EEG Acquisition and Signal Preprocessing

EEG data were acquired with a 32 Ag/AgCl electrode cap referenced to the average of left and right mastoids and a SynAmps amplifier (Compumedics Neuroscan Ltd, Australia) digitized at a 1000-Hz sampling rate. The contact impedance between all electrodes and the skin was maintained at $ < 5~\text{k}\Omega $. Before further data analysis, the raw EEG signals were bandpass filtered between 0.5 and 50 Hz using a zero-phase FIR filter (EEGLAB toolbox [45]). Then, filtered data were downsampled from 1000 Hz to 250 Hz to reduce computational complexity. In addition, independent Component Analysis (ICA) was applied to decompose the EEG signals into independent time courses presumably arising from distinct brain sources [46]–[48]. Components related to eye movements and blinking were identified by visual inspection and removed. And each channels’ related components were selected to reconstruct EEG channels’ signals which preserve the original signal feature as much as possible. After which, the trials relating to the three different MI conditions were extracted as epochs.

### D. Quantification of ERD and ERS

Since the mu (alpha) ERD and ERS are the main index for MI-BCI [30], we used this index to confirm whether these three tasks can evoke the same MI phenomena. To compute the time course of ERD in alpha band (8 to 13 Hz), a similar procedure was adopted as reported in the literature [49], [50]. These procedure involves the following four steps: (1) bandpass filtering of all event-related MI trials, (2) squaring of the amplitude samples to obtain power samples, (3) averaging of power samples across all trials, and (4) averaging samples over time to smooth the data and reduce the variability. The ERD/ERS was here defined as percentage power decrease (ERD) or power increase (ERS) in relation to the average of first two seconds fixation stage (~2~0 s) before the motor imagery stage (0~5 s). The power in the interested frequency band in the period after the event is given by ‘A’, whereas baseline is given by ‘R’. ERD% is expressed as follows:

\begin{equation*} \mathrm {ERD}\left ({\% }\right)=\frac {A-R}{R}\times 100\tag{1}\end{equation*}

where both A and R are EEG power averages of all trials in the specific MI tasks.

### E. Feature Extraction and Classification of Motor Imagery

After EEG signal preprocessing, a typical signal processing pipeline is used for feature extraction and classification approaches for MI-BCI verification and applications. Currently, common spatial pattern (CSP) is one of the most common feature extraction methods used in MI BCI [51], [52]. CSP is a spatial filtering method used to minimize the variance of one class and maximize the variance of another class simultaneously [53]–[57]. Therefore, it is sensitive to binary differences, such as the left- and right-motor imagery [51], [52]. However, it is not easy to identify the optimal frequency range for each subject. To solve this problem, the sub band CSP (SB-CSP) method [58], a filter bank composed of different interested sub bands as inputs, was applied in this study. The equations are as follows:

Two windows from the multivariate signal are represented as $S_{1}$ with size $\left ({n,t_{1} }\right)$ and $t_{1}$ samples and $S_{2}$ with size $\left ({n,t_{2} }\right)$ and $t_{2}$ samples. To make the rate of variance maximum between two windows, the CSP algorithm defines component $W^{T}$and is represented as follows:

\begin{equation*} \mathrm {W=}{\mathrm {max}}_{w}\frac {\left \|{ wS_{1} }\right \|^{2}}{\left \|{ wS_{2} }\right \|^{2}}\tag{2}\end{equation*}

The solutions are obtained by calculating the two covariance matrices below:

\begin{equation*} R_{1}=\frac {S_{1}S_{1}^{T}}{t_{1}}~and~R_{2}=\frac {S_{2}S_{2}^{T}}{t_{2}}\tag{3}\end{equation*}

When computing the generalized eigenvalue decomposition of the two matrices, eigenvectors are obtained for matrix as $\mathrm {E=}\left [{ e_{1}\ldots e_{n} }\right]$ and sorted by the descending diagonal matrix D of eigenvalues $\left \{{\lambda _{1}\ldots }\right.\left.{ \lambda _{n} }\right \} $as follows:

\begin{equation*} E^{T}R_{1}E=D{andE}^{T}R_{2}E=I_{n}\tag{4}\end{equation*}

Then, $W^{T}$ will be $w=e_{1}^{T}$.

Subsequently, linear discriminant analysis (LDA) is applied to identify the EEG signals in each sub-band spectrum [59]. LDA is a well-known binary classification method that can project data in a new space using $\mathrm {y= }w^{T}x$ to minimize the within class scatter and maximize the scatter between classes [56]. The accuracy of CSP combined the LDA approach for MI BCI classification had been discussed in our previous work [60], and detailed information can be found in those studies. In this study, the raw EEG data are filtered into three frequency bands including alpha (8 to 13 Hz) and beta (13 to 30 Hz) for adopting the SB-CSP and LDA to investigate the accuracy of each MI task.

### F. Functional Connectivity Estimated By Transfer Entropy

Brain connectivity analysis, particularly functional and effective connectivity, represents one of the most promising approaches to investigate the brain operation of MI and improve MI-BCI performance [2], [42], [61]. Transfer entropy (TE) is the best-known method in this area, which naturally incorporates directional and dynamical information because it is inherently asymmetric and based on transition probabilities [44]. In previous works, TE has demonstrated its robustness against volume conduction and its effectiveness in investigating nonlinear interactions between brain areas [44], [61]–[63].

TE is a model-free measure based on information theory and can be used to calculate the amount of directed information transmission between two systems [64]. TE is deduced from information and conditional transition probabilities between any two processes evolving in time. Let $X=\left \{{x_{1},x_{2},\cdots,x_{n} }\right \}$ and $Y=\left \{{y_{1},y_{2},\cdots,y_{n} }\right \}$ be two processes that represent two interacting systems (time series) that can be approximated by stationary Markov processes. Thereupon, using a delay embedded vector ( $x_{t}^{d}=\left \{{x_{t},x_{t-\tau },x_{t-2\tau },\cdots,x_{t-\left ({d-1 }\right)\tau } }\right \})$, we could easily reconstruct the full state space of the processes of interest. The dimension of the embedding space was d, and the delay was $\tau $ [44]. According to the assumption, the system S could be approximated by a stationary Markov process of order d. Then, the transition probability of the system was described as follows: $P(x_{i+1}\mathrm {\vert }x_{t}^{d})$. The entropy rate of system X is defined as the amount of additional information required to represent the value of the additional state. All previous states of the systems are known and can be computed as follows:

\begin{align*} H\left ({x_{t+u}\thinspace \vert \thinspace x_{t}^{d}}\right) \!=\!-\sum \limits _{x_{t+u},x_{t}^{d}} {P\left ({x_{i+u}\thinspace \vert \thinspace x_{t}^{d}}\right) \log {P\left ({x_{t+u}\thinspace \vert \thinspace x_{t}^{d}}\right)}} \\\tag{5}\end{align*}

The conditional probability $P\left ({x_{i+u}\thinspace \vert \thinspace x_{t}^{d}}\right) $ is calculated from the joint probabilities $P\left ({x_{i+u}\thinspace \vert \thinspace x_{t}^{d}}\right) =P(x_{i+u},x_{t}^{d}\mathrm {)/}P(x_{t}^{d})$. The probabilities $P\left ({\ast }\right)$ are estimated via kernel estimation or the k-nearest neighbor approach [65]. To measure the amount of information transferred from process Y to process X, the entropy rate of system X is defined as the amount of additional information required to represent the value of the additional state. As noted for all previous states of the systems, we deduce this state as follows:

\begin{align*}&\hspace {-.5pc} H\left ({x_{i+u}\thinspace \vert \thinspace {x_{t}^{d},y_{t}^{m}}}\right) =-\sum \limits _{x_{t+u},x_{t}^{d}} P\left ({x_{t+u}\thinspace \vert \thinspace {x_{t}^{d},y_{t}^{m}}}\right) \log \\&\qquad\qquad\qquad\qquad\qquad\qquad\qquad \times {P\left ({x_{i+u}\thinspace \vert \thinspace {x_{t}^{d},y_{t}^{m}}}\right)}\tag{6}\end{align*}

If two different processes were not dependent, there is no transfer of information, and it would cause $H\left ({x_{i+u}\thinspace \vert \thinspace x_{t}^{d}}\right) \mathrm {= }H\left ({x_{i+u}\thinspace \vert \thinspace {x_{t}^{d},y_{t}^{m}}}\right) $, where the state of X only depends on d states of X. According to previous work, it used Kullback divergence or mutual information to compute a measure of deviation from this generalized Markov property. The information flow from Y to X, which is called transfer entropy $\mathrm {TE(Y \to X)}$, was computed as follows [64]:

\begin{align*} TE\left ({Y\to X }\right)=\sum {P\left ({x_{t+u},x_{t}^{d},y_{t}^{m} }\right)} \log \frac {P\left ({x_{t+u}\thinspace \vert \thinspace {x_{t}^{d},y_{t}^{m}}}\right)}{P\left ({x_{t+u}\thinspace \vert \thinspace x_{t}^{d}}\right)} \\\tag{7}\end{align*}

where u was the prediction time. The joint probabilities $P\left ({\ast }\right)$ can be estimated by kernel density estimation or the k-nearest neighbor approach [62]. Conditional transition probabilities were determined from the embedding vector to sum the TE value. To estimate the TE value, it is necessary to reconstruct the embedding space of the data [63]. The embedding space was reconstructed by $x_{t}^{d}=\left \{{ x_{t},x_{t-\tau },x_{t-2\tau },\cdots,x_{t-\left ({d-1 }\right)\tau } }\right \}$. The dimension d was obtained by an effective search algorithm, and the delay $\tau $ was determined by Cao’s criterion [66]. The equation can be rewritten as follows:

\begin{align*}&\hspace {-.5pc} TE\left ({Y\to X }\right)=H\left ({x_{t}^{d},y_{t}^{m} }\right)-H\left ({x_{t+u}{,x}_{t}^{d},y_{t}^{m} }\right) \\&\qquad\qquad\qquad\qquad\qquad\qquad +H\left ({x_{t+u}{,x}_{t}^{d} }\right)-H\left ({x_{t}^{d} }\right)\tag{8}\end{align*}

TE indicates directional information flow and was inherently asymmetric; thus, $TE\left ({Y\to X }\right)\mathrm {\ne }TE\left ({X\to Y }\right)$. However, when the two signals of processes are independent, $TE\left ({Y\to X }\right)=TE\left ({X\to Y }\right)\mathrm {=0}$.

The estimation of parameters and the calculation of TE were performed using TRENTOOL (Version 2.0.4) [63]. In this study, prediction time u was set to 1 and K was set to 4 for the k-nearest neighbor approach as suggested by a previous study [65]. The window(T) of Theiler correction was set to 1 to remove the autocorrelation effect from the density estimation.

In this study, the estimation of effective connectivity between the selected channels have been conducted over various MI conditions and different EEG frequency bands, alpha (8-13Hz), beta (14-30Hz), and gamma (31-50Hz) bands, for both left- and right-hand motor imagery. The TE mean values and standard deviations of any two of the selected channels’ connectivity pairs (source to sink) across all subjects have been calculated for each MI task under three frequency bands when they performed left- and right-hand imagery.

### G. Statistical Analysis

The TE values of the MI stage in three EEG frequency bands were normalized by subtracting the mean baseline (fixation stage) for the motor imagery of both hands under each MI condition. Therefore, in this study, the normalized TE values are relative-TE values, which represent the changes with respect to the baseline period. Nine electrodes (F3, Fz, F4, C3, Cz, C4, P3, Pz and P4) were selected to investigate brain connectivity during the motor imagery stage of the three MI conditions. For further analysis, subjects’ TE values of the connectivity pairs in each MI task were averaged and tested using the Wilcoxon signed rank test with an FDR correction (p<0.05). Given the complexity of the MI task, two comparisons (rMI vs rmMI; bcMI vs rmMI) were taken in this study to test whether the rotation movement and bilateral coordination can evoke more brain interaction when performing single hand motor imagery tasks.

## III. Experimental Results

### A. ERD/ERS for Three MI Tasks

**FIGURE 2.**
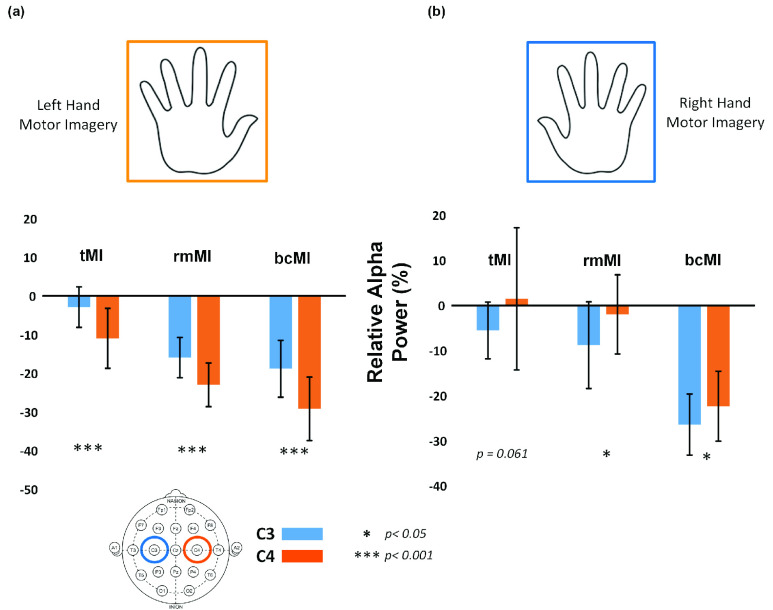
Mu (alpha) ERD comparisons of contralateral and ipsilateral channels under left and right-hand motor imagery (corresponding to a and b) for three MI tasks, including the traditional MI task (tMI), rotation movement MI task (rmMI) and bimanual coordination MI task (bcMI). The stars (*) represent the statistical significant levels (Wilcoxon signed rank test), * for p<0.05 and *** for p<0.001. The y-axis is alpha power change relative baseline (%).

Fig. 2 shows the comparisons of averaged ERD of contralateral and ipsilateral channels under left and right hand motor imagery for three MI tasks (tMI, rmMI,and bcMI). The Wilcoxon signed rank test with FDR-adjusted was used to compare the difference between the ERD at the two channels (C3 and C4) under the motor imagery stage of both hands for the three MI tasks. As shown in Fig. 2, the alpha power was significantly suppressed at the contralateral channel as compared to the ipsilateral channel for motor imagery of both hands under all MI tasks (p<0.05), except for the right hand imagery in the tMI condition (p=0.061). The results demonstrate that the slightly complex video feedback employed in these MI conditions did not disturb the MI performance, and all participants were able to successfully perform the assigned motor imagery, which were reflected in the alpha desynchronization changes at the motor area.

### B. Classification Accuracy of Three MI Tasks

To confirm whether MI-BCI performance is influenced by the rotation movement (rmMI) and both hands representation (bcMI) designed in this study, the CSP and LDA were applied to test the classification accuracy of these three MI conditions.

**FIGURE 3.**
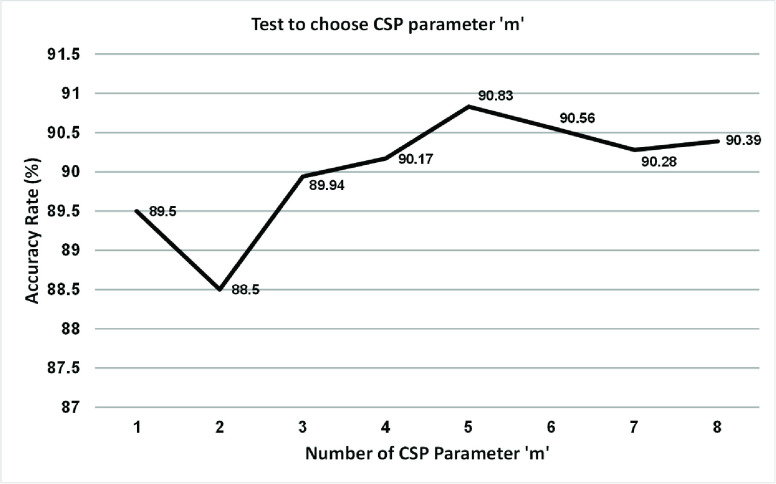
The selection of parameter comparisons for CSP.

The optimal value for the parameter ‘m’ in the CSP algorithm was determined by testing values in the range of 1 to 8 to maximize the classification accuracy. For each value of m, after the CSP projection, 10-fold cross validation was applied to data 100 times. The test results for all subject under the three MI conditions were averaged and these results are shown in Fig. 3 Based on these results, ‘m’ = 5 achieves the highest accuracy value (90.83). Thus, m = 5 is selected for the CSP projections under every MI condition.

**FIGURE 4.**
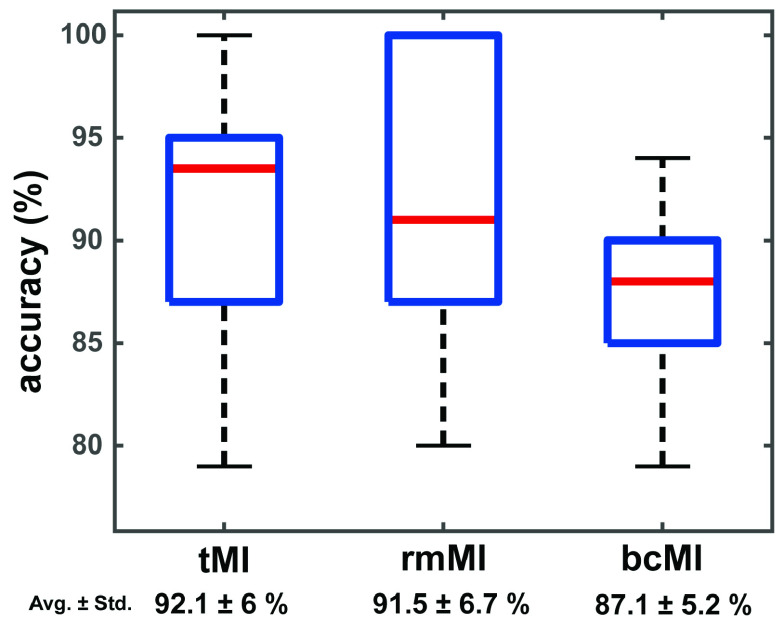
Box plot of classification accuracy distributions of tMI, rmMI, and bcMI (Wilcoxon rank sum test, p < 0.05).

After extracting the EEG features by CSP, LDA was applied to perform the classification for all the three MI conditions. Here, 10-fold cross validation was applied 100 times. Fig. 4 shows the accuracy distributions of three MI tasks. Although the highest accuracy rate (92.1 ± 6) is noted for tMI condition, the rmMI and bcMI conditions still achieve high classification accuracies (91.5 ± 6.7 and 87.1 ± 5.2). In Fig. 2, tMI and rmMI seem to be more lateralized than bcMI, but in Fig. 4, the accuracy of MI tasks has no significant difference among the three tasks. Therefore, the rotation movement and the bi-hand presentations of visual feedback do not impede the operation and the classification of motor imagery. These results demonstrate that the participants were able to follow the experiment instructions and perform motor imagery of the assigned single hand successfully in all three MI tasks without any distraction.

The above accuracy results of LDA classification with CSP filters among three MI tasks indicate that the newly proposed MI conditions can also evoke similar responses as compared to the traditional MI condition as all participants were able to correctly perform the motor imagery under all conditions without any prior experience.

### C. Functional Connectivity Under Various MI Conditions

**FIGURE 5.**
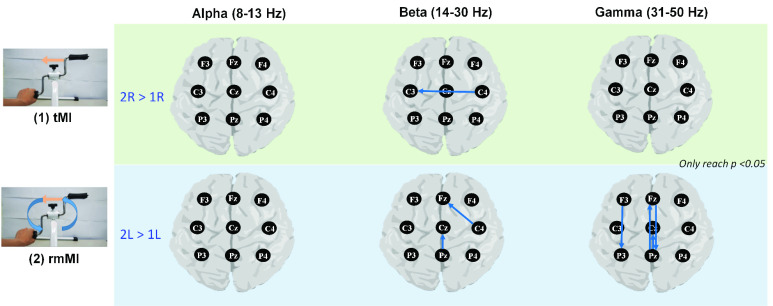
Pairwise comparisons of TE topographic connectivity between rmMI and tMI tasks. Only statistical tests that achieved significance (p<0.05) were retained. 2R > 1R and 2L > 1L mean the significantly increased amount of connectivity of the rmMI task under right-hand motor imaginary and left-hand motor imagery relative to the tMI task, respectively.

**FIGURE 6.**
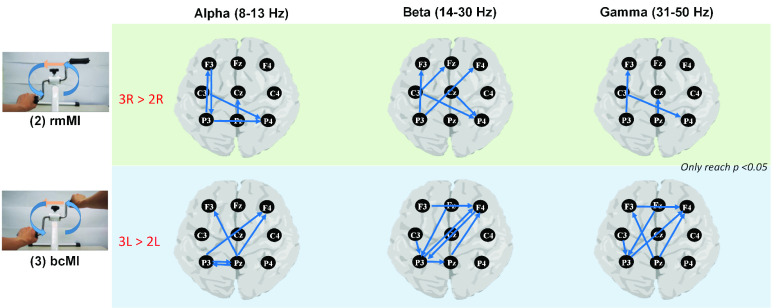
Pairwise comparisons of TE topographic connectivity between bcMI and rmMI tasks. Only statistical tests that achieved significance (p<0.05) were retained. 3R > 2R and 3L > 2L mean the significantly increased amount of connectivity of the bcMI task under right-hand motor imaginary and left-hand motor imagery relative to the rmMI task, respectively.

In Fig. 5 and Fig. 6, the TE mean values of 72 connectivity pairs (source to sink) from nine selected channels for each MI task have been evaluated under three frequency band (alpha, beta, and gamma bands).

The pairwise connectivity measures that achieve statistical significance (FDR-adjusted p<0.05) during tMI and rmMI conditions are shown in Fig. 5. Fig. 6 shows the statistically significant pairwise connectivity measures during rmMI and bcMI conditions. These results illustrate minimal differences among three frequency bands in the comparisons between the tMI and rmMI conditions. In the contrast, the bcMI shows more significant and complex connectivity than the rmMI for all three bands, especially in the left hand imagery.

As shown in Fig. 5, no significant differences in alpha bands were found between the rmMI and tMI. However, rmMI exhibited increased information flow of the beta band from C4 to C3 (right-hand imagery), Pz to Cz (left-hand imagery) and C4 to Fz (left-hand imagery). The difference was also shown in the gamma band analysis for left-hand imagery only, including from Pz to Cz, Pz to Fz, Fz to Pz and F3 to P3. Since the alpha and beta are the basic brain indices for motor imagery, the rotation movement of rmMI does not seem to influence the operation of motor imagery at the motor area. The space information of visual rotation exhibits a feed-forward pattern from the parietal to central area with the beta rhythm and can be used to adjust the position of the imaged hand via fronto-parietal gamma synchronization.

In Fig. 6, the bcMI task demonstrated an increase amount of connectivity compared to the rmMI task. With the EEG alpha features, bcMI task infers directional flow from Pz to Cz, P3 to P4, P3 to F3, C3 to P4 and F3 to P3 when performing right-hand imagery tasks and from P3 to F4, Pz to P3, Pz to F3 and Pz to F4 in the left-hand MI condition. In the beta analysis, observed significant brain connectivity changes are found from P3 to F3, P3 to F4, C3 to P4, C3 to Fz and Cz to P4 in the right hand MI and from P3 to Pz, P3 to F4, Pz to F4, C3 to P3, F3 to F4 and F4 to P3 for imaging the left hand. Similar results are found in the gamma band. Compared to the rmMI, the bcMI task exhibited increased information flow from P3 to Fz, Pz to Cz and C3 to P4 for right-hand MI and from P3 to F4, Pz to F3, Pz to F4, C3 to P3, F3 to F4 and Fz to P3 for the left-hand imagery condition. Significant differences are noted when performing the right- and left-hand imagery tasks in this comparison.

## IV. Discussion and Conclusion

In this study, novel MI-BCI, bimanual and rotated motor imagery is proposed to explore brain connectivity when performing ipsilateral motor imagery tasks. The basic MI phenomena were supported in all three conditions based on the ERD changes in the contralateral alpha frequency band, and the results were classified by CSP and LDA.

The subjects performed the ipsilateral motor imagery task with bilateral or rotation visual feedback. Based on these MI criteria, the bcMI-BCI can evoke increased brain connectivity, especially when performing the left-hand imagery task.

As shown in Fig. 6, in the right-hand imagery task, the contralateral parietal site was strongly connected to the contralateral frontal region across all frequency bands and provided two-way communication via alpha rhythm. Moreover, left motor area showed increased information flow to the ipsilateral parietal region with all three frequency EEG features. In the left-hand imagery task, with the exception of the space information of rotation movement that was sent from the parietal to both lateral frontal regions that are related to motor action planning, the ipsilateral parietal region had a strong connection with the contralateral frontal areas across all frequency bands. However, bidirection flows were noted in the beta and gamma analysis. A more interesting finding is the interaction between the bilateral frontal electrodes via beta and gamma rhythms.

Based on our experimental results, this study supports the following conclusions:

(1) mutual communication occurs between the prefrontal area (F3 and F4), which indicates that the nonimagined hand (ipsilateral site) and imagined hand (contralateral site) interact with each other for motor preparation and bilateral arm coordination after receiving visual feedback;
(2) communication occurs from the parietal region to bilateral frontal area (Pz to F3 and F4), which indicates that rotation of the hand-bike kept providing the spatial location information of each hand for motor planning. Because the neural patterns are influenced by the function of handedness, less connectivity in right hand condition could be due to all participants are the same right-handed and can image more efficiently [67].

In summary, this study demonstrates the dynamic connectivity when using bcMI and could be used as the index to investigate the reorganization and reconnection of motor cortex with the MI rehabilitation approach.

## V. Limitations

Although the current study validated the brain connectivity while executing bcMI to investigate the reorganization and reconnection of the motor cortex with the MI rehabilitation approach, the present demonstration still has some limitations.

First, the connectivity of bimanual and rotated motor condition could be still influenced by the methodological limitation. Therefore, the weighted effect of rotation movement or solely the bimanual component should be further investigated with bimanual static imagery.

Second, although the study has yielded consistent results across the subjects participating in the experiment, more testing samples and comparison tests were still needed to confirm effectiveness and robustness.

Third, our findings exhibit the ordinary people’s brain connectivity of bcMI as the index; however, for further MI rehabilitation, the future work of the study should collect more data from the patient subjects, such as the stroke patients, for validation.
